# Acupotomy Therapy for Shoulder Adhesive Capsulitis: A Systematic Review and Meta-Analysis of Randomized Controlled Trials

**DOI:** 10.1155/2019/2010816

**Published:** 2019-12-13

**Authors:** Jianyu You, Fengyun Yang, Naigang Liu, Nana Tang, Ting Fang, Fushui Liu, Zhongyong Liu, Hongwu Xie

**Affiliations:** ^1^College of Acupuncture-Moxibustion and Tuina, Jiangxi University of Traditional Chinese Medicine, Nanchang, China; ^2^College of Clinical Medicine, Jiangxi University of Traditional Chinese Medicine, Nanchang, China; ^3^China-Japan Friendship Hospital, Beijing, China

## Abstract

**Objective:**

Acupotomy therapy is widely used for pain management. However, the efficacy of acupotomy on shoulder adhesive capsulitis (SAC) is still uncertain. The aim of this study was to determine the effectiveness and safety of acupotomy therapy for SAC.

**Methods:**

We searched seven electronic databases to collect randomized controlled trials (RCTs) of acupotomy for SAC published before April 2019. A meta-analysis was performed according to the Cochrane systematic review method by using RevMan 5.3 software.

**Results:**

A total of eight RCTs involving 501 patients were enrolled. Meta-analysis showed that acupotomy was significantly better than the control group in debasing the Visual Analogue Scale (VAS) score (MD = −0.97, 95% CI = [−1.49, −0.45], *P*=0.0003) and improving the Constant–Murley Score (CMS) (MD = 8.46, 95% CI = [1.04, 15.87], *P*=0.03), and there was no significant difference in adverse events (OR = 1.24, 95% CI = [0.34, 4.52], *P*=0.74) between the two groups.

**Conclusion:**

Acupotomy therapy is an effective and safe treatment for SAC, and this treatment can be recommended for the management of SAC. Due to the low quality and small sample size of the included studies, more rigorously designed RCTs with high quality and large-scale are recommended in future.

## 1. Introduction

Shoulder adhesive capsulitis (SAC), also known as “frozen shoulder,” is a common condition of the shoulder. Clinically, it can be evolved from shoulder impingement syndrome, manifested by pain, stiffness, and limited range of motion (ROM) of the affected shoulder [1–3], and it can further lead to sleep deprivation, anxiety, and even disability, which seriously affect the patient's daily life and occupational activities [4]. According to statistics, the prevalence of SAC is estimated at 2% to 5% of the general population. The majority of patients diagnosed with SAC is women between the ages of 40 and 60, and the prevalence of patients with diabetes and hypothyroidism is significantly increased [5, 6]. SAC imposes enormous economic pressure on individuals and society. In UK, the National Health Service (NHS) costs at least £44.1 million to £110.3 million based on a single general practitioner consultation in each case [7]. Traditionally, it has been suggested that SAC is often self-limiting, and the symptoms can be completely eliminated without treatment. However, recent research evidence challenges this theory, and studies have shown that as many as 40% of patients report persistent symptoms and movement restriction beyond 3 years and up to 15% of patients suffering from permanent disability [6].

Although a subset of patients progress to permanent disability, first-line therapies for this condition are often conservative. Conventional treatments include nonsteroidal anti-inflammatory drugs (NSAIDs), short-term oral corticosteroids, steroid injections, and physiotherapy [1]. Although SAC has multiple treatment options, most of these interventions are often accompanied by significant side effects [8, 9]. Excitingly, the clinical study of acupotomy treatment of SAC showed promising results. Acupotomy invented by Professor Zhu Hanzhang is a new type of therapy that combines Traditional Chinese Medicine meridian theory with modern surgical techniques [10]. It absorbs modern anatomy and pathology theory as well as sterility and anesthesia techniques [11]. It uses acupotomy as a treatment tool to perform appropriate operations on the tissue of local lesions, such as cutting and peeling, penetrating, and shoveling. Acupotomy treatment can effectively eliminate adhesions, contractures, and relieves the tension of soft tissue to restore the normal function of the tissue [12, 13], and it has the characteristics of small wound, less complications, high safety, low cost and high treatment efficiency [14]. Therefore, it is widely used clinically for musculoskeletal diseases characterized by chronic soft tissue damage and bone and joint diseases including SAC [13].

Although acupotomy has been used in clinical treatment for only a few decades, there are currently more than one hundred thousand acupotomy practitioners in China [15]. In addition, previous systematic reviews and meta-analyses have reported the efficacy of acupotomy [16–18]. However, the primary outcomes of the efficacy criteria used in most studies have not been internationally recognized, and the acupotomy group is primarily a mixed intervention. It consists of acupotomy combined with other therapies, so we cannot objectively evaluate the efficacy of treating the SAC with acupotomy alone. Therefore, we conducted a new systematic review to clarify the effectiveness of a single acupotomy in the treatment of SAC and provide evidence-based medical advice for future research and treatment.

## 2. Methods

### 2.1. Search Strategy

Two reviewers (Jianyu You and Ting Fang) independently performed a comprehensive literature search from multiple electronic databases, including EMBASE, PubMed, the Cochrane Library, the China National Knowledge Infrastructure (CNKI), the WanFang databases, the Chinese Biomedical Literature Database (CBM), and the China Science and Technology Journal Database (VIP) from inception to April 1, 2019. The terms used for the search were “acupotomy OR acupotomology OR needle knife OR needle scalpel” and “adhesive capsulitis OR scapulohumeral periarthritis OR frozen shoulder OR periarthritis humeroscapularis” in each database. Then, we browsed the abstracts and full-text articles, respectively, and picked the eligible studies in line with the inclusion criteria.

### 2.2. Inclusion and Exclusion Criteria

Studies that met the following criteria were included: (1) types of studies: only RCTs of acupotomy therapy for SAC were included. RCTs were published in English or Chinese, with the full-text available; (2) types of participants: participants who met the diagnostic criteria for SAC; (3) types of interventions: acupotomy therapy which only included acupotomy alone, regardless of different acupoints or needle materials. Since functional exercise is an important part of the rehabilitation of SAC, acupotomy with or without functional exercise will also be included; (4) types of control groups: the control group will receive an internationally recognized therapy such as conventional pharmacological therapies or steroid injections. No treatment, placebo, and acupuncture will also be included (since there is no study report on sham acupotomy and acupotomy originating from the innovation of acupuncture, acupuncture will also be included); and (5) types of outcome measures: studies measured at least one of the following authoritative indicators: Visual Analogue Scale (VAS), Constant–Murley score (CMS), and adverse events.

Studies with the following situations were excluded: (1) Non-RCTs; (2) lack of definitive diagnostic criteria; (3) wrong intervention measures (interventions in RCTs have other treatments besides acupotomy and functional exercise); (4) unusable data; and (5) repeated publication.

### 2.3. Data Extraction

Two reviewers (Jianyu You and Ting Fang) independently extracted relevant data from the eligible studies, and any disagreement was settled through discussion with a third reviewer (Fushui Liu). The extracted study data mainly included the first author, year of publication, study location, baseline characteristics for participants, sample size, intervention, duration of intervention, follow-up, outcome measurement indexes, and adverse events.

### 2.4. Quality Assessment

Two reviewers (Jianyu You and Ting Fang) independently evaluated the risk of bias in each included literature according to the Cochrane risk of bias assessment tool, and discrepancies were resolved by discussion. The contents include: (1) random sequence generation; (2) allocation concealment; (3) blinding of participants and personnel; (4) blinding of outcome assessment; (5) incomplete outcome data; (6) selective reporting; and (7) other sources of bias.

### 2.5. Statistical Analysis

RevMan 5.3 software was applied in this meta-analysis. For continuous variables (VAS and CMS), when outcomes were measured by the same scale, results were reported as mean differences (MDs) with 95% confidence intervals (CIs); when outcomes were measured by different scales, results were reported as standardized mean differences (SMDs) with 95% CI. Categorical data (adverse events) was calculated with the odds ratio (OR) and 95% CI. We defined *P* < 0.05 as statistically significant between studies. Heterogeneity was evaluated by Chi-squared test and Higgins *I*^2^ test; when *I*^2^ ≤ 50% and *P* > 0.10, the fixed effect model was used; otherwise, the random effect model was applied. Considering the clinical heterogeneity of the type of control groups, we performed subgroup analysis based on different control groups. Sensitivity analysis was used to assess the impact of the included trials on the final outcome. Publication bias was estimated by funnel plot analysis if sufficient studies were included.

## 3. Results

### 3.1. Literature Search Results

**Figure 1:**
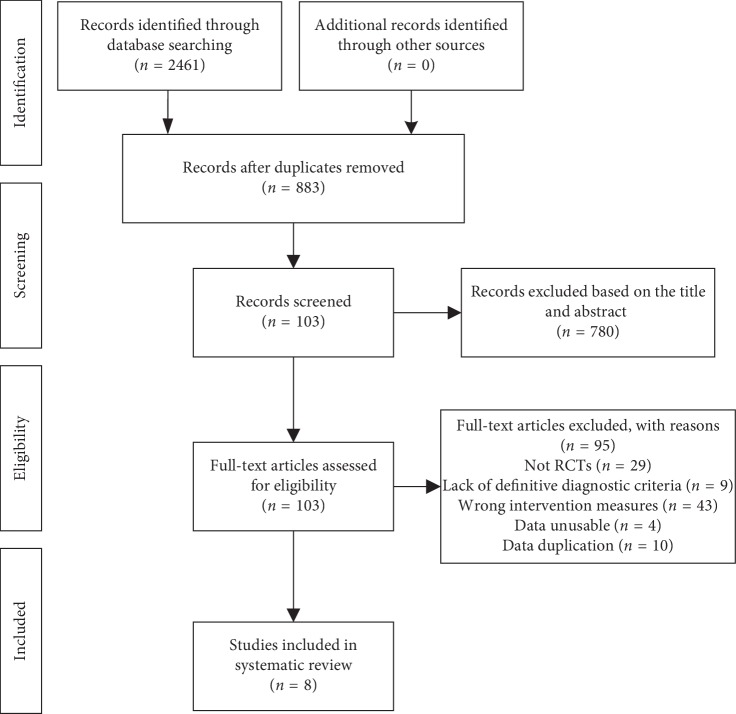
Flow diagram of the study.

A total of 2,461 potentially relevant studies were identified at the initial search. It remained 883 studies after we excluded 1,578 duplicates with EndNote X7 software. Then, after reading the titles and abstracts, 780 studies were eliminated. Finally, 8 studies [19–26] were eligible and included in the systematic review. The whole process of study selection is shown in Figure 1.

### 3.2. Basic Characteristics of Eligible Studies

The basic characteristics of all included studies were provided in Table 1. All studies were published between 2009 and 2017. All of the studies were conducted in China and published in Chinese with a total of 501 participants: 251 in experiment groups and 250 in control groups. The sample size ranged from 39 to 78 participants. There were five studies [19, 20, 24–26] which compared acupotomy with acupuncture: one study [23] compared acupotomy with no treatment, one study [22] compared acupotomy with oral drugs, and one study [21] compared acupotomy with steroid injection.

### 3.3. Quality Assessment

**Figure 2:**
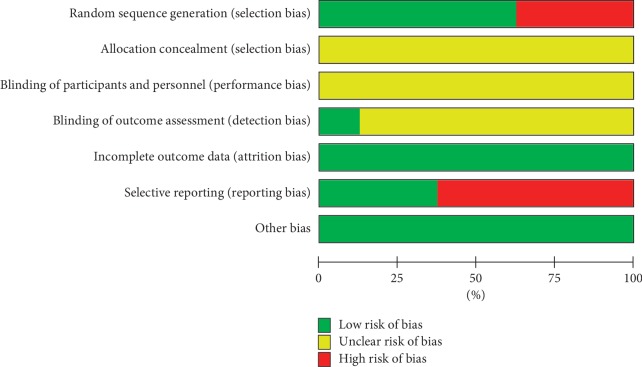
Risk of bias graph: review authors' judgments about each risk of bias item presented as percentages across all included studies.

**Figure 3:**
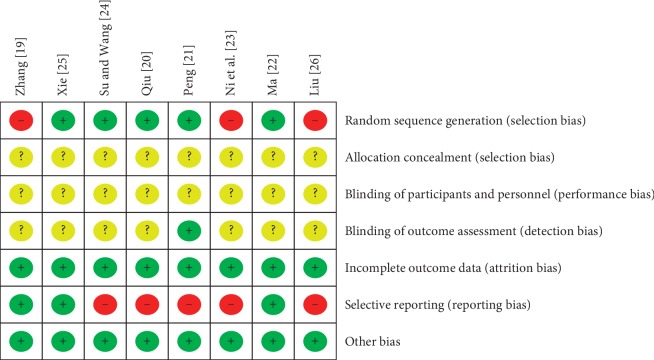
Risk of bias summary: review authors' judgments about each risk of bias item for each included study.

Among the 8 included studies, 3 studies [20, 22, 25] used a random number table to generate random sequence, and two studies [21, 24] used computer program to generate random sequence. The rest studies only mentioned “random.” No study mentioned the use of allocation concealment. Due to the inherent characteristics of acupotomy manipulations, it is difficult to conduct blinding, and only one study [21] reported the blinding details about outcome assessment. One study [21] reported dropout numbers and reasons, and the other studies reported no missing data. One study [19] reported no adverse events, two studies [22, 25] reported adverse events, and the rest did not report any details about adverse events. The risk of bias (ROB) results are shown in Figures 2 and 3.

### 3.4. VAS Score

**Figure 4:**
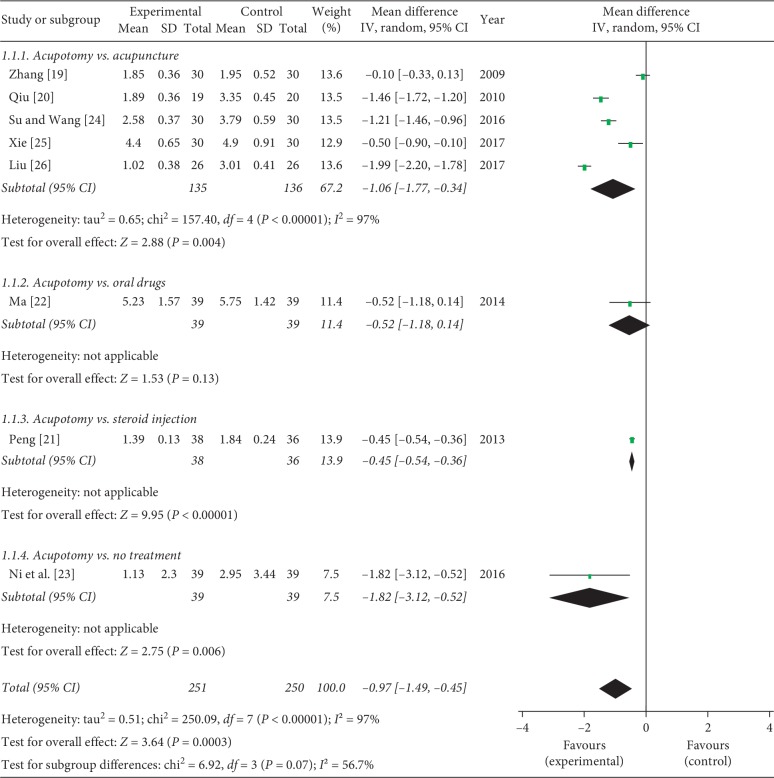
Meta-analysis for the VAS score of acupotomy versus the control group.

All the studies evaluated pain intensity by using the VAS score. Five studies compared acupotomy with acupuncture, one study compared acupotomy with no treatment, one study compared acupotomy with oral drugs, and one study compared acupotomy with steroid injection. Data extracted showed obvious heterogeneity among these RCTs (*P* < 0.00001, *I*^2^ = 97%), a random effects model was used, and our pooled results showed acupotomy could further relieve pain compared with the control group (MD = −0.97, 95% CI = [−1.49, −0.45], *P*=0.0003). Subgroup analysis also showed that acupotomy is statistically significantly better than acupuncture (MD = −1.06, 95% CI = [−1.77, −0.34], *P*=0.004), heterogeneity (*P* < 0.00001, *I*^2^ = 97%), steroid injection (MD = −0.45, 95% CI = [−0.54, −0.36], *P* < 0.00001), and no treatment (MD = −1.82, 95% CI = [−3.12, −0.52], *P*=0.006). However, there was no statistically significant difference between acupotomy and oral drugs based on one study (MD = −0.52, 95% CI [−1.18, 0.14], *P*=0.13) (Figure 4).

### 3.5. CMS

**Figure 5:**
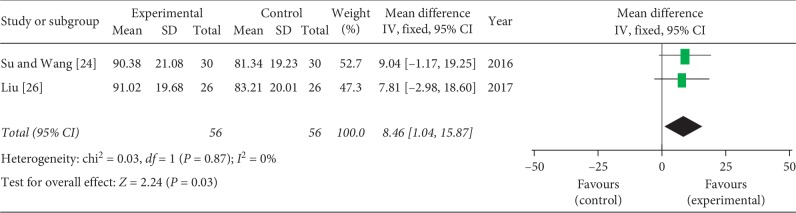
Meta-analysis for CMS of acupotomy versus control group.

Two studies [24, 26] compared acupotomy with acupuncture and reported the CMS to evaluate shoulder joint function. Analysis of data from CMS showed no heterogeneity (*P*=0.87, *I*^2^ = 0%), and the fixed-effects model showed that acupotomy could further improve shoulder function compared with the acupuncture group (MD = 8.46, 95% CI = [1.04, 15.87], *P*=0.03) (Figure 5).

### 3.6. Adverse Events

**Figure 6:**
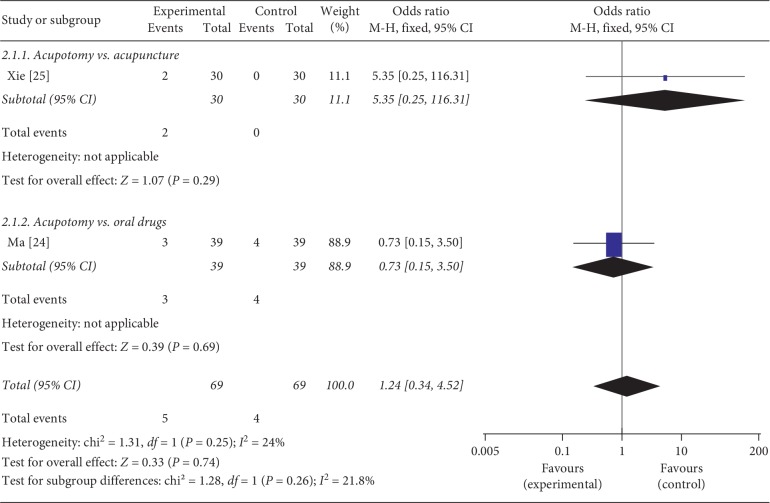
Meta-analysis for adverse events of acupotomy versus control group.

Two studies [22, 25] reported mild adverse events during the treatment, such as local bruising or soreness at needle site and nausea. One study compared acupotomy with acupuncture, and one study compared acupotomy with oral drugs. No heterogeneity was found between the two studies (*P*=0.25, *I*^2^ = 24%), and the fixed-effects model showed that no statistical difference in adverse events between acupotomy and control group (OR = 1.24, 95% CI = [0.34, 4.52], *P*=0.74). In addition, subgroup analysis also showed no statistically significant differences between acupotomy and acupuncture (OR = 5.35, 95% CI = [0.25, 116.31], *P*=0.29), as well as acupotomy and oral medications (OR = 0.73, 95% CI = [0.15, 3.50], *P*=0.69) (Figure 6).

### 3.7. Heterogeneity and Sensitivity Analysis

There was considerable heterogeneity (*I*^2^ = 97%) in the comparison of acupotomy versus acupuncture on the VAS score. We performed sensitivity analysis by excluding potential heterogeneous studies one by one, and sensitivity analyses indicated that the results of meta-analysis were stable. However, the heterogeneity was not resolved and may be caused by selection of acupoints, depth of insertion, manipulation frequency. The sensitivity analyses of other outcomes were not conducted due to the small number of included studies.

### 3.8. Publication Bias

Due to the insufficient number of included studies (fewer than 10 studies), we did not conduct analysis of reporting bias by funnel plot.

## 4. Discussion

Shoulder adhesive capsulitis is a common shoulder joint disease, which is generally divided into three stages: pain stage, frozen stage and thawing stage [27]. However, the exact pathogenesis of SAC remains controversial. The most commonly accepted hypothesis states that inflammation initially occurs within the joint capsule and synovial fluid. The inflammation is followed by reactive fibrosis and adhesions of the synovial lining of the joint [28]. The initial inflammation of the capsule leads to pain, and the capsular fibrosis and adhesions lead to a decreased range of motion. In addition, a recent review suggested that SAC may begin as an immunological response which escalates to an inflammatory synovitis, eventually leading to fibrosis of the capsule [4, 29].

Acupotomy is a new minimally invasive treatment method combining acupuncture and scalpel. With the theory of acupuncture as the guiding ideology, it can not only achieve the stimulation effect of acupuncture, but also play the role of cutting and peeling of scalpel [11]. The biggest difference between acupuncture and acupotomy is that the end of acupuncture is the tip of the needle, and the end of acupotomy is the blade of the needle. Therefore, compared with the acupuncture, acupotomy can also perform various operations on the tissue of local lesions, such as cutting, peeling, penetrating, and shoveling [14]. Although acupotomy is also called the “small needle knife,” the small needle knife is not the real meaning of “knife.” Like acupuncture, acupotomy is also percutaneously inserted into the body. Therefore, acupotomy can effectively avoid skin incision and minimize anatomical damage during treatment [11, 30]. In addition, recent studies provide laboratory-based evidence that acupotomy could modulate inflammatory response to alleviate pain by regulating a variety of inflammatory cytokine levels, such as substance P (SP), 5-hydroxytryptamine (5-HT), interleukin-1*β* (IL-1*β*), interleukin-10 (IL-10), tumor necrosis factor-*α* (TNF-*α*), and transforming growth factor-*β* (TGF-*β*) [10, 31]. Simultaneously, acupotomy also has a good regulating effect on superoxide dismutase (SOD) and total antioxidant capacity (T-AOC) in serum and local muscles [32], and histopathological studies have found that acupotomy can reduce synovial thickening and tendon fibrosis, improve local pathological state, and promote tissue repair [33].

In our current study, we included 8 RCTs that compared acupotomy with acupuncture, oral drugs, steroid injection, and no treatment. With respect to reducing pain, we used the VAS score to indicate the intensity of pain. In this study, the scales used for the VAS scores in the 8 RCTs we included were consistent, so we used the mean difference (MD) of the VAS scores to report the results. Our pooled analysis indicated that acupotomy was more effective than acupuncture, steroid injection, and no treatment. However, there was no statistically significant difference between acupotomy and oral drugs based on one study. With respect to improving the CMS, the scales used for the CMS in the 2 RCTs we included were consistent, so we used the mean difference (MD) of the CMS to report the results. Our pooled analysis indicated that acupotomy was more effective than acupuncture. In this meta-analysis, only two studies reported relevant adverse events. The combined data showed no significant difference in adverse reactions between the acupotomy group and control group. In addition, subgroup analysis also suggests the same result. Therefore, we can carefully recommend that acupotomy is as safe as the control group for SAC. Based on the findings of our included studies, we propose that acupotomy is an effective and safe therapy for SAC. In addition, because acupotomy treatment is a closed procedure, there are strict requirements for the operator. The operator's perception of the disease and its familiarity with the anatomical location are critical to the therapeutic effect and operation security. Therefore, the safety of acupotomy is often questioned. Excitingly, the latest study reports a safer acupotomy method [34] that uses ultrasound guidance rather than experience to locate the lesion and the operating site of the needle. In the direction of the ultrasound, we can easily observe the location of the lesion and the operating conditions of the needle. Therefore, ultrasound guidance can not only improve the efficacy of acupotomy but also improve the safety of acupotomy, which has broad application prospects in the future [35].

However, the current meta-analysis has several limitations as follows: first, the insufficient number of included studies and the small sample sizes may lead to imprecise evidence in our study. Second, the methodological quality of most studies is low; no study mentioned the use of allocation concealment, and only one study reported the blinding details that might limit the accuracy of the conclusions of this meta-analysis. Third, there was significant heterogeneity in our study. Subgroup analysis and sensitivity analyses were used to explore the source of heterogeneity. However, the heterogeneity has not been resolved. We considered that this heterogeneity may stem from methodological bias and differences in acupoint selection, the frequency and duration of treatment. Last, only a few studies have reported details of adverse events and follow-ups; therefore, the safety and long-term effects of acupotomy for SAC remains to be further explored. Given the above limitations, it is recommended to use more rigorous large-scale and well-designed RCTs to provide higher quality evidence and to evaluate the efficacy of acupotomy in the treatment of SAC.

## 5. Conclusions

The results of our current systematic review and meta-analysis suggested that acupotomy therapy is an effective and safe treatment for SAC, and it can be recommended for the management of SAC. However, our conclusions have many limitations, such as insufficient number of included studies, low methodological quality, and heterogeneity of results. Therefore, more large-scale and high-quality RCTs are needed to further investigate the efficacy of acupotomy for SAC.

## Figures and Tables

**Table 1:** Characteristics of included studies.

Study	Study location	Sample size (male/female)	Interventions	Treatment period	Outcomes	Adverse events
Zhang [19]	Hunan, China	AG: 10/20	AG: acupotomy	AG: once per week for 4 weeks	VAS	No
		CG: 11/19	CG: acupuncture	CG: once a day for 28 days, 30 min		

Qiu [20]	Fujian, China	AG: 7/12	AG: acupotomy	AG: once per week for 3 weeks	VAS	NR
		CG: 9/11	CG: acupuncture	CG: once every two days for 20 days, 30 min		

Peng [21]	Heilongjiang, China	AG: 20/18	AG: acupotomy + FE	AG: Once per week for 3 weeks	VAS	NR
		CG: 16/20	CG: steroid injection + FE	CG: NR		

Ma [22]	Beijing, China	AG: 15/24	AG: acupotomy + FE	AG: once every 6 days, a total of 3 times, FE once a day for 14 days	VAS	Not serious, mild
		CG: 19/20	CG: oral drugs (naproxen sodium) + FE	CG: Three times a day for 14 days, FE once a day for 14 days	AE	

Ni et al. [23]	Hubei, China	AG: 16/23	AG: acupotomy + FE	AG: once per week for 3 weeks, FE once a day for 21 days	VAS	NR
		CG: 14/25	CG: FE	CG: FE once a day for 21 days		

Su and Wang [24]	Gansu, China	AG: 16/14	AG: acupotomy	AG: once per week for 3 weeks	VAS	NR
		CG: 15/15	CG: acupuncture	CG: six times a week for 3 weeks, 30 min	CMS	

Xie [25]	Guangxi, China	AG: 10/20	AG: acupotomy + FE	AG: once per week for 4 weeks, 20 to 30 minutes, FE three times a day for 28 days	VAS	Not serious, mild
		CG: 11/19	CG: acupuncture + FE	CG: five times a week for 4 weeks, FE three times a day for 28 days	AE	

Liu [26]	Shandong, China	AG: 26	AG: acupotomy	AG: NR	VAS	NR
		CG: 26	CG: acupuncture	CG: NR	CMS	

Abbreviations: AE, adverse events; AG, acupotomy group; CG, control group; CMS, Constant–Murley score; FE, functional exercise; NR, not reported; VAS, The visual analogue scale.
